# Draft Genome Sequences of 12 Monophasic Salmonella enterica subsp. *enterica* Serotype Typhimurium 1,4,[5],12:i:− Strains Isolated from Wild Griffon Vultures in Eastern Spain

**DOI:** 10.1128/MRA.00570-19

**Published:** 2019-10-17

**Authors:** Clara Marin, Giuseppe D’Auria, Llúcia Martínez-Priego, Francisco Marco-Jiménez

**Affiliations:** a Instituto de Ciencias Biomédicas. Departamento de Producción Animal, Sanidad Animal y Ciencia y Tecnología de los Alimentos, Facultad de Veterinaria, Universidad Cardenal Herrera-CEU, CEU Universities, Alfara del Patriarca, Valencia, Spain; b Servicio de Secuenciación y Bioinformática, Fundación para el Fomento de la Investigación Sanitaria y Biomédica de la Comunidad Valenciana (FISABIO-Salud Pública), Valencia, Spain; c CIBER en Epidemiología y Salud Pública (CIBEResp), Madrid, Spain; d Instituto de Ciencia y Tecnología Animal, Universitat Politècnica de València, Valencia, Spain; University of Maryland School of Medicine

## Abstract

Monophasic Salmonella enterica subsp. *enterica* serovar Typhimurium is one of the most common zoonotic pathogens. *Salmonella* species reside in a wide variety of hosts, including wild animals. Thus, we report here the genome sequences of 12 monophasic *S.* Typhimurium strains isolated from healthy wild vultures to gain better insight into their epidemiology and host-pathogen interactions.

## ANNOUNCEMENT

*Salmonella* spp. stand out as some of the most common causes of human bacterial food poisoning (1). Specifically, Salmonella enterica subsp. *enterica* serovar Typhimurium represented 21.8% of all reported serovars of confirmed human cases in 2016 in the European Union (1). Wild birds are vectors in the dissemination of livestock and human pathogens (2), including *Salmonella* spp. (3). Nevertheless, the number of wildlife species acting as reservoirs, amplifiers, and disseminators is unknown (4). *Salmonella* spp., including *S*. Typhimurium, have been isolated from vultures in several studies (2, 5, 6). Wild vulture monophasic *S.* Typhimurium strains displayed genomic DNA (gDNA) fingerprinting patterns similar to those observed in *Salmonella* strains from pig farms, suggesting that pig farms introduce *Salmonella* infection into vultures at supplementary feeding stations (5). Hence, genome analysis of multiple isolates of this *Salmonella* lineage should help to evaluate the potential risk to wildlife and the environment. To that end, we have sequenced the genomes of 12 isolates of monophasic *S.* Typhimurium strains isolated from wild vultures (Table 1).

**TABLE 1:** Monophasic Salmonella enterica subsp. *enterica* serotype Typhimurium 1,4,[5],12:i:− strains isolated from wild griffon vultures in eastern Spain

Isolate	Genome size (bp)	No. of reads	No. of contigs	Coverage (×)	*N*_50_ length (bp) (no. of contigs)	GC content (%)	ENA read accession no.	ENA assembly accession no.
Vulture-STm-CyP-1	5,162,468	806,314	67	63.47	461,225 (4)	49	ERR3385856	SAMEA5530637
Vulture-STm-CyP-2	5,265,851	669,207	84	51.01	442,846 (3)	50	ERR3385857	SAMEA5530638
Vulture-STm-CyP-3	5,336,318	862,984	66	69.78	423,460 (4)	50	ERR3385858	SAMEA5530639
Vulture-STm-CyP-4	5,026,433	1,175,547	58	91.76	461,244 (4)	50	ERR3385859	SAMEA5530640
Vulture-STm-CyP-5	4,559,258	1,270,926	57	101.19	492,138 (3)	50	ERR3385860	SAMEA5530641
Vulture-STm-CyP-6	5,344,085	945,925	75	74.92	416,712 (4)	49	ERR3385861	SAMEA5530642
Vulture-STm-CyP-7	5,026,635	909,679	56	71.52	423,380 (4)	50	ERR3385862	SAMEA5530643
Vulture-STm-CyP-8	5,076,141	666,708	62	50.92	423,466 (4)	50	ERR3385863	SAMEA5530644
Vulture-STm-CyP-9	5,025,060	798,412	56	59.62	461,235 (4)	50	ERR3385864	SAMEA5530645
Vulture-STm-CyP-10	5,350,630	929,782	68	73.59	492,062 (4)	50	ERR3385865	SAMEA5530646
Vulture-STm-CyP-11	5,025,905	835,164	57	64.13	461,239 (4)	50	ERR3385866	SAMEA5530647
Vulture-STm-CyP-12	5,026,886	946,564	58	74.40	422,950 (4)	50	ERR3385867	SAMEA5530648

### DNA extraction and sequencing.

Genomic DNA was isolated using a MagNA Pure LC DNA isolation kit III (Bacteria, Fungi; catalog number 03264785001). DNA libraries were generated using the Illumina Nextera XT library prep kit (catalog number FC-131-1024) and starting from 0.2 ng/μl of purified gDNA measured by a Qubit double-stranded DNA (dsDNA) high-sensitivity (HS) assay kit (catalog number Q32851). Sample multiplexing was performed using a Nextera XT index kit set C (catalog number FC-131-2003). Libraries were then sequenced using a 2 × 300-bp paired-end run (MiSeq reagent kit v3, catalog number MS-102-3001) on an Illumina MiSeq sequencer.

### Bioinformatics analysis.

Default parameters were used for all software, unless otherwise specified. The obtained raw sequencing data were quality checked using the PRINSEQ-lite program v0.20.4 (7), eliminating reads shorter than 50 bp, trimming for a minimum mean Q30 from the right side in a window of 20 bp. Residual adaptor-related sequences were eliminated using Trimmomatic v0.36 (8).

The obtained reads were mapped against the Salmonella enterica subsp. *enterica* serovar Typhimurium SL1344 genome (GenBank accession number NC_016810) using the BWA-MEM algorithm (http://bio-bwa.sourceforge.net/). Unmapped reads were filtered out using SAMtools suite v1.8 (9) and assembled *de novo* using the MIRA program v3 (10). Contigs obtained from mapping against the reference genome have been extracted using BEDTools v2.24.0 (11), with the contig order following the mapping. *De novo* assembled contigs have been concatenated at the end of the assembly in order to collect complete genomic information. The final data sets were then annotated using Prokka v1.13 (12) to search for coding sequences (CDSs) annotated against the proper reference genome GenBank annotations, High-quality Automated and Manual Annotation of microbial Proteomes (HAMAP) v201701.18 (13), and Pfam v31.0 (14) databases. Table 1 summarizes the main genomic features.

The genomes ranged from 4.5 to 5.3 Mb in size, as described for other *Salmonella* strains (15). Sequencing generated an average G+C content of 49.78%, which is similar to that reported previously for other *Salmonella* isolates (15). The number of contigs per assembly for each isolate ranged between 56 and 84. These genomes should allow for a detailed comparison of the attributes of monophasic *S.* Typhimurium isolates from wild vultures with those from other wild birds, livestock, and the environment, and with isolates contaminating the food chain.

### Data availability.

This whole-genome shotgun project has been deposited in the European Nucleotide Archive (ENA) under the project accession number PRJEB31693 and the sample accession numbers SAMEA5530637 to SAMEA5530648 (Table 1).
